# Maize Inoculation with *Azospirillum brasilense* Ab-V5 Cells Enriched with Exopolysaccharides and Polyhydroxybutyrate Results in High Productivity under Low N Fertilizer Input

**DOI:** 10.3389/fmicb.2017.01873

**Published:** 2017-09-26

**Authors:** André L. M. Oliveira, Odair J. A. P. Santos, Paulo R. F. Marcelino, Karina M. L. Milani, Mónica Y. A. Zuluaga, Claudemir Zucareli, Leandro S. A. Gonçalves

**Affiliations:** ^1^Departamento de Bioquímica e Biotecnologia, Centro de Ciências Exatas, Universidade Estadual de Londrina, Londrina, Brazil; ^2^Departamento de Agronomia, Centro de Ciências Agrárias, Universidade Estadual de Londrina, Londrina, Brazil; ^3^Departamento de Biotecnologia, Escola de Engenharia de Lorena, Universidade de São Paulo, Lorena, Brazil

**Keywords:** surface response methodology, alternative fertilizers, nitrogen-fixing bacteria, inoculant effectiveness, plant growth-promoting bacteria

## Abstract

Although *Azospirillum* strains used in commercial inoculant formulations presents diazotrophic activity, it has been reported that their ability to produce phytohormones plays a pivotal role in plant growth-promotion, leading to a general recommendation of its use in association with regular N-fertilizer doses. In addition, a high variability in the effectiveness of *Azospirillum* inoculants is still reported under field conditions, contributing to the adoption of the inoculation technology as an additional management practice rather than its use as an alternative practice to the use of chemical inputs in agriculture. To investigate whether the content of stress-resistance biopolymers would improve the viability and performance of *Azospirillum* inoculants when used as substitute of N-fertilizers, biomass of *A. brasilense* strain Ab-V5 enriched in exopolysaccharides (EPS) and polyhydroxybutirate (PHB) was produced using a new culture medium developed by factorial mixture design, and the effectiveness of resulting inoculants was evaluated under field conditions. The culture medium formulation extended the log phase of *A. brasilense* cultures, which presented higher cell counts and increased EPS and PHB contents than observed in the cultures grown in the OAB medium used as control. An inoculation trial with maize conducted under greenhouse conditions and using the biopolymers-enriched Ab-V5 cells demonstrated the importance of EPS and PHB to the long term bacterial viability in soil and to the effectiveness of inoculation. The effectiveness of liquid and peat inoculants prepared with Ab-V5 cells enriched with EPS and PHB was also evaluated under field conditions, using maize as target crop along different seasons, with the inoculants applied directly over seeds or at topdressing under limiting levels of N-fertilization. No additive effect on yield resulted from inoculation under high N fertilizer input, while inoculated plants grown under 80% reduction in N fertilizer showed yields at levels compared to fully fertilized plants, regardless the inoculation method. The presented data highlights the feasibility to partially substitute the N-fertilizer demand in non-legume crops using high-quality inoculant formulations, prepared with diazotrophic bacteria enriched with stress-resistance biopolymers that confer increased viability an effectiveness to the bacterial cells.

## Introduction

Inoculation technology with plant growth-promoting bacteria (PGPB) has been presented worldwide as an important tool for reaching sustainability in agriculture due to its low environmental and production costs compared with industrial inputs. However, different from symbiotic relationships, where plant-bacteria interactions have been widely exploited and are relatively well understood, the associative interactions driven by PGPB are discreet and elicited by factors that have only recently started to be clarified (Carvalho et al., 2016; Zhou et al., 2016). It is not difficult to realize that the broad adoption of PGPB inoculation as regular agricultural practice is somehow impaired by the lack of scientific knowledge regarding the ecology, physiology and biochemistry of associative plant-bacteria interactions. Although feasible, the replacement of chemical inputs in commercial agriculture with bioproducts developed from and based on the rational exploitation of plant-microbe natural relationships, such as nutrient-provider bacteria in substitution for soluble fertilizers, or biocontrol agents in substitution for pesticides, remains a major challenge (Baez-Rogelio et al., 2017). In this sense, efforts to strengthen inoculation technology in non-leguminous crops with PGPB need to incorporate a broader understanding of the determinants of bacterial rhizocompetence and competitiveness necessary to successfully achieve a plant-PGPB interaction. In the same way, one must consider the physiological status of the inoculated microorganisms such that high viability is maintained under adverse conditions found in soil and/or during storage. Such challenges are magnified if one considers the identification of PGPB with high biotechnological potential in distinct phylogenetic clusters, and the low probability of finding conditions to produce high-quality inoculants that can be universally applied for any PGPB (Bashan et al., 2014).

Commercial inoculants carrying PGPB are generally available as dry or liquid formulations of different organic and/or inorganic materials, which may be prepared with cells grown in a liquid culture medium or via the direct use of bacterial broth for producing liquid inoculants, or obtaining dehydrated cells that may be incorporated in a solid or liquid carrier (Bashan, 1998; Malusá et al., 2012; Cassán and Díaz-Zorita, 2016). Liquid inoculants simplify both the industrial production and the field application, although compared with solid formulations, such as peat- or polymer-based inoculants, bacteria in liquid inoculants appear to be more sensitive to stressful conditions and can exhibit decreased viability when used on seeds or soil (Herrmann and Lesueur, 2013). Effectiveness of PGPB inoculants has been improved by immobilization of inoculant cells in polymeric carriers, such as alginate and starch foam (Bashan et al., 2016; Marcelino et al., 2016). Thus, the actual demand is for improved liquid inoculant formulations, which are replacing peat-based inoculants and currently comprise ~80% of doses sold for soybean crops in Brazil (Hungria et al., 2015). While the technical criteria required to produce high-performance peat-based inoculants are well defined, these same criteria are treated as industrial secrets or proprietary information for liquid inoculants. Even considering the presence of high-performance *Azospirillum*-based liquid inoculants on the market, the information regarding the composition of these inoculants is mostly limited to that presented on the product label, which increases the difficultly of conducting a thorough scientific analysis of the role of each ingredient in the formulation and its effects on the final quality of the product applied in the field (Bashan et al., 2014). This may be best exemplified by inoculant formulations using the PGPB *Azospirillum brasilense*, which are prepared from different bacterial strains and are available in a wide variety of commercial products worldwide; variations in their performance under field conditions are still reported.

The diazotroph *A. brasilense* is considered a model PGPB, and a great amount of information regarding the physiology of its growth and development has been published (Fendrik et al., 1995; Cassán et al., 2015). However, it is unclear when this available knowledge is in fact applied in the inoculant industry. Quality control assessments of agricultural inoculants are commonly defined by the presence of contaminants and the population density of viable cells in the final product throughout its shelf-life, and determining any information about the physiological state of the inoculant PGPB strain in a commercial product is not required. To consider inoculant production solely in terms of the yield of microbial biomass from a specific strain has the potential to produce poor-quality inoculants, which plays against the broad adoption of PGPB inoculation as an alternative to conventional agronomical practices. To implement this new paradigm in modern agriculture, commercial inoculant formulations should be produced with bacterial cells at a high population density and in high physiological state to enable them to face adverse conditions that occur both during storage (shelf life) and at the time of its use (on seeds and in soil) (Herrmann and Lesueur, 2013; Bashan et al., 2014; Bashan and de-Bashan, 2015). It is not difficult to realize that the broad adoption by the industrial and regulatory agencies of the available PGPB physiology knowledge, as well as the implementation of research studies aimed to better understand the physiological characteristics for which no such knowledge is yet available, should result in better inoculants with higher field performance.

At least in Brazil, the inoculation of cereals with diazotrophic PGPB, such as maize inoculation with *A. brasilense*, is considered an additional practice and its adoption occurs under the application of regular amounts of N fertilizer that can exceed 200 kg ha^−1^. Concerns leading to the distrust of the natural nitrogen inputs provided by diazotrophic PGPB substituting at least part of the nitrogen demand in commercial crops reflect the lack of scientific information available for the commercial inoculant formulations. In addition, there is a huge variability in inoculation efficiency as a result of the use of low-quality inoculants (Bashan, 1998; Herrmann and Lesueur, 2013; Vassilev et al., 2015). Maize inoculants are mainly applied over seeds before sowing, which is an additional practice that presents risks of lowering the germination rate if the seeds are mechanically damaged during this process. Furthermore, inoculating seeds places the bacteria in contact with the pesticides and agrochemicals commonly found covering commercial seeds, which are then sown close to the fertilizers applied in the soil. Intending to determine whether conditioning the PGPB *A. brasilense* Ab-V5 to accumulate high amounts of polyhydroxybutyrate (PHB) and exopolysaccharides (EPS) during its growth leads to an improved inoculant performance in the field, a culture medium was developed, and growth conditions were defined to lead this bacterium to increase its biopolymer contents. The importance of the cellular content of PHB and EPS on improving the viability of *A. brasilense* was evaluated by a short-period assay carried out under greenhouse conditions. Bacterial biomass produced under the conditions defined so on was used to produce liquid inoculants, which were applied on seeds or topdressing in maize plants; the results of these treatments were compared with the performance of a peat inoculant applied on seeds. We demonstrate that this approach resulted in highly efficient liquid inoculants under the tested conditions and that topdressing inoculation is a suitable alternative management for PGPB inoculants. The results also indicate that a significant reduction in the amount of N fertilizer applied to maize crops is possible without decreasing crop productivity.

## Materials and methods

### Bacterial strain

The bacterial strain *A. brasilense* Ab-V5, originally isolated from maize plants in Brazil and registered in the Brazilian Ministry of Agriculture, Livestock and Food Supply (MAPA) to produce commercial inoculants for maize, wheat and rice in Brazil (Hungria et al., 2010), was used as a model for developing a culture medium and for performing the inoculation trials with maize. This strain is deposited at the “Culture Collection of Diazotrophic and Plant Growth Promoting Bacteria” of Embrapa Soja, Londrina, Paraná, Brazil.

### Culture medium

To develop the culture medium, a pre-inoculum of *A. brasilense* was prepared in 25 mL of DYGS liquid medium (Rodrigues Neto et al., 1986), placed in 250 mL Erlenmeyer flasks. The pre-inoculum was shaken at 150 rpm and 28 ± 2°C for 24 h; then, the bacterial population density was determined by direct counts in a Neubauer chamber and used to inoculate 50 mL of different culture medium formulations in 250 mL Erlenmeyer flasks. The initial population was 1 × 10^4^ cells mL^−1^, following incubation in a rotary shaker at 150 rpm and 28 ± 2°C for 24 h. A 2^4^-factorial design was applied to study the effects of glycerol (*X*_1_), crystal sugar (*X*_2_), yeast extract (*X*_3_) and potassium monohydrogen phosphate (*X*_4_) on *A. brasilense* growth and EPS production, resulting in 17 different formulations with three replicates at the central point, as presented in Table 1. The components and concentration ranges adopted to define the composition of the culture medium was based on previous studies that resulted in the simplification of the tryptone-yeast extract-glucose (TYG) medium (data not shown). Each culture medium formulation was prepared according to the factorial design, supplemented with 0.1 mL L^−1^ micronutrient solution (g L^−1^: H_3_BO_3_, 1.4; ZnSO_4_.7H_2_O, 1.2; MnSO_4_.H_2_O, 1.18; Na_2_MoO_4_.2H_2_O, 1.0; CuSO_4_.5H_2_O, 0.04) and adjusted to pH 6.5.

**Table 1:** Mixture composition of the culture medium formulations based on a 2^4^-factorial design for evaluating the effect of glycerol, sucrose, yeast extract and potassium monohydrogen phosphate on EPS and biomass production (colony forming units, CFU counts) by *Azospirillum brasilense* Ab-V5.

**Run**	**Coded variable**				**Real variable (%** ***w/v*****)**				**Responses**	
	***X*_1_**	***X*_2_**	***X*_3_**	***X*_4_**	**Glycerol**	**Sucrose**	**YE**	**K_2_HPO_4_**	**Log CFU mL^−1^**	**EPS (g L^−1^)**
1	−1	−1	−1	−1	3.0	1.0	1.0	0.05	9.17	1.656
2	1	−1	−1	−1	10.0	1.0	1.0	0.05	9.18	0.701
3	−1	1	−1	−1	3.0	5.0	1.0	0.05	9.11	0.612
4	1	1	−1	−1	10.0	5.0	1.0	0.05	6.00	0.136
5	−1	−1	1	−1	3.0	1.0	5.0	0.05	9.40	1.107
6	1	−1	1	−1	10.0	1.0	5.0	0.05	8.98	0.475
7	−1	1	1	−1	3.0	5.0	5.0	0.05	8.77	0.333
8	1	1	1	−1	10.0	5.0	5.0	0.05	6.00	0.109
9	−1	−1	−1	1	3.0	1.0	1.0	0.15	9.11	0.607
10	1	−1	−1	1	10.0	1.0	1.0	0.15	9.70	0.294
11	−1	1	−1	1	3.0	5.0	1.0	0.15	8.42	1.475
12	1	1	−1	1	10.0	5.0	1.0	0.15	9.38	1.055
13	−1	−1	1	1	3.0	1.0	5.0	0.15	7.24	0.103
14	1	−1	1	1	10.0	1.0	5.0	0.15	9.15	0.657
15	−1	1	1	1	3.0	5.0	5.0	0.15	9.37	1.970
16	1	1	1	1	10.0	5.0	5.0	0.15	9.45	1.229
17	0	0	0	0	5.0	2.0	2.5	0.10	9.43	1.161
18	0	0	0	0	5.0	2.0	2.5	0.10	9.41	1.116
19	0	0	0	0	5.0	2.0	2.5	0.10	9.44	1.203

*Responses (EPS and CFU counts) were recorded after 24 h of incubation in a rotary shaker at 150 rpm and 28 ± 2°C*.

A mathematical model was created to describe the relationships between the independent variables CFU (colony forming units) and EPS (coded as *Y*), and the independent variables glycerol (*X*_1_), crystal sugar (*X*_2_), yeast extract (*X*_3_), and potassium monohydrogen phosphate (*X*_4_). The experimental data designed were matched to the following second-order polynomial equation:

(1) $$\begin{array}{l} Y=\beta_{0}+\overset{4}{\underset{i=1}{\sum }}\beta_{i}X_{i}+\overset{4}{\underset{i=1}{\sum }}\;\underset{i\neq j}{\sum }\beta_{ij}X_{i\;}X_{j}+\beta_{1234\;}X_{1}X_{2}X_{3}X_{4\;} \end{array}$$

where *Y* represents the CFU of *A. brasilense* Ab-V5 mL^−1^ of culture media or the EPS production in g L^−1^, and β_0_, β_i_, and β_ij_ are the constant, linear and interaction coefficient estimates, respectively. The coded variables were applied to fit the model and determine the linear and combined effects of the independent variables on the dependent variables. To evaluate the influence of the independent variables on the dependent variables, the CFU counts and the EPS concentration in the culture medium formulations were determined as follows. The CFU in each formulation tested was assessed by the drop plate method (Miles and Misra, 1938) from aliquots of 1.0 mL of each culture medium collected after the growth period (24 h at 28 ± 2°C in a rotary shaker at 150 rpm), serially diluted in a sterilized saline solution (0.85 % NaCl in distilled water, *w/v*) and inoculated (0.02 mL of dilutions 10^−5^–10^−8^) in Petri dishes containing DYGS solid medium (15 g L^−1^ agar-agar). The colony counts were performed after the plates were incubated for an additional 18–24 h at 28 ± 2°C. The analysis was performed in triplicate, and the results are expressed as CFU of *A. brasilense* per mL of the culture medium tested (CFU mL^−1^). The EPS concentration in each culture medium formulation was determined according to a method describe by Mozzi et al. (1996). Briefly, aliquots of 10 mL were centrifuged (12,000 g × 30 min at 4°C) to precipitate the bacterial cells. The cell-free supernatant of each culture medium formulation was collected, mixed with 30 mL of cold ethanol and incubated for 24 h at 4 ± 2°C to precipitate the soluble EPS. Precipitated EPS was solubilized in 2 mL of distilled water and dialyzed against distilled water for 24 h at 4 ± 2°C (molecular weight cutoff of 13 kDa; Sigma-Aldrich, Germany) to eliminate residual salts and sugars. The EPS was lyophilized, the quantity was expressed as g EPS L^−1^ culture medium, and the analysis was performed in triplicate.

### Physiological parameters of *A. brasilense* Ab-V5 grown in a culture medium optimized for high EPS levels and CFU counts

Based on the results of the factorial design presented above, a culture medium formulation was selected for further investigation to determine the growth kinetics, biomass production, EPS concentration in the cell-free supernatant, amount of intracellular PHB and bacterial viability in comparison with those of the OAB medium (modified NFb by increasing its buffering capacity and supplementing micronutrients, a nitrogen source and yeast extract; Bashan and de-Bashan, 2015) which was used as a control. To accomplish this, a pre-inoculum of *A. brasilense* Ab-V5 was prepared in DYGS liquid medium as stated above and used to inoculate 250 mL of both the selected and OAB culture media in 1,000 mL Erlenmeyer flasks. The initial *A. brasilense* population was 1 × 10^4^ cells mL^−1^, and the bacterial growth kinetics were determined by CFU counts measured at intervals of 3, 6, 12, 18, 24, 36, 48, and 60 h after inoculation. Growth rate and generation time were calculated according to methods described by Bashan et al. (2011). The biomass production was determined by weighing the cells precipitated from 10 mL samples of each formulation at 12, 36, and 60 h after inoculation. After sampling, each culture was centrifuged (12,000 g × 15 min at 4°C), and the pellet was washed twice in 5 mL of sterilized saline solution following each centrifugation step. The final pellet was lyophilized and used to determine the biomass concentration in the culture medium and to quantify the level of PHB. The amount of EPS produced in each culture medium was determined as its concentration in the cell-free supernatant, as presented in the previous section. PHB was extracted and quantified according to the method described by Law and Slepecky (1961), with a few modifications. Briefly, the cell biomasses from the selected and control culture media obtained as described above were suspended in 12 mL of sodium hypochlorite (5.25%, v/v) and incubated for 2 h at 40 ± 2°C. This solution was centrifuged (12,000 g × 15 min at 4°C), and the supernatant was discarded. Subsequently, the precipitated material was washed with distilled water (10 mL, manual shaking and centrifugation at 12,000 g × 15 min at 4°C) and cold acetone (10 mL, manual shaking and centrifugation at 12,000 g × 15 min at 4°C). Then, the precipitate was suspended in 3 mL of ethanol and heated to 80°C to evaporate the ethanol. The hydrolysis of PHB to crotonic acid was achieved by adding 1.0 mL of concentrated sulfuric acid and incubating the samples at 90°C for 30 min. After the samples cooled, the absorbance was measured at 235 nm using H_2_SO_4_ as a blank, and PHB was quantified by using the following relation: PHB (mg/L) = Abs (235 nm) × 0.064516. Each experimental analysis was performed in triplicate.

### Inoculation trials

The experimental designs described below were intended to evaluate the performance of *A. brasilense* Ab-V5 inoculants in promoting the growth, productivity and nitrogen nutrition of commercial maize genotypes, which are continuously developed by seed companies which launch several new genotypes on the market at each crop season. To avoid a significant influence of the genotype-genotype compatibility effect between the inoculant bacteria and the maize genotype over the results, different commercial maize seeds were used in each experiment described below, according to its relevance of use in commercial crops and availability in the local market. The agronomical characteristics of maize hybrids used are presented in the Supplementary Table 1.

#### Greenhouse assay

A completely randomized experimental design was conducted to investigate the protective effect of the cellular content of PHB and EPS on *A. brasilense* viability when exposed to the soil. Pots filled with 2 kg of an unsterilized substrate prepared by mixing sand and oxisol with high clay content (78 %) in a proportion of 2:1 (*v*:*v*) were placed in a greenhouse and constituted the experimental units. The substrate presented the following: pH (in H_2_O), 5.1; H + Al (cmol_c_ dm^−3^), 9.28; K (cmol_c_ dm^−3^), 0.63; Ca (cmol_c_ dm^−3^), 4.1; Mg (cmol_c_ dm^−3^), 1.5; Al (cmol_c_ dm^−3^), 0.32; P (mg dm^−3^), 13.0; and organic matter (%), 2.23. One day before the experiment started, pots received 400 mL of tap water, and the day the experiment started a further watering with 50 mL of tap water were applied to each pot. Prior to initiate the experiment, the inoculants were set down directly over the moisten substrate using a micropipette with sterile tips, 2–3 cm below the surface without mixing the inoculant with the substrate, in order to leave Ab-V5 cells in the soil before sowing the pots with the maize seeds.

Inoculants were prepared from a pre-inoculum of *A. brasilense* Ab-V5 in DYGS medium and used to inoculate 50 mL of the OAB and the selected culture media, in 250 mL Erlenmeyer flasks with an initial population of 1 × 10^4^ cells mL^−1^. *A. brasilense* Ab-V5 was incubated on both culture media at 28 ± 2°C in a rotary shaker set to 150 rpm, with interruption of culturing after 12, 36, or 60 h of growth. In total, six different inoculants were obtained: three from Ab-V5 grown in OAB medium and three from Ab-V5 grown in the selected formulation, reflecting different EPS and PHB cellular contents as determined by the different growth periods of time. For each of these inoculants, the bacterial population density was normalized to a final concentration of 1 × 10^6^ cells mL^−1^ by dilutions in the respective fresh culture media. To assure that the diluted cultures carried equivalent populations of viable cells, the normalization of all inoculants was performed just before its use by determining the number of viable cells on each culture with the aid of the LIVE/DEAD BacLight kit (ThermoFisher Scientific), used according to the manufacturer instructions and visualized in an AxioVision epifluorescence microscope (Carl Zeiss).

The experimental design comprehended 20 treatments with five replicates, distributed as follow. A set of pots added with 1 mL of fresh OAB or fresh selected media, that were sown in the same day of substrate treatment (OAB control and FORM15 control). Another set of pots distributed in six treatments, which received 1 mL of inoculants from both culture media at each culturing time (OAB-12 h, OAB-36 h, OAB-60 h, FORM15-12 h, FORM15-36 h, and FORM15-60 h) also sown in the same day of substrate treatment (sowing at day 0). A third set of pots distributed as the previous one (six treatments), but which were sown 4 days after the substrate had received the inoculants (sowing at day 4). A final set of pots distributed in the same six treatments, but sown 8 days after the substrate had received the inoculants (sowing at day 8). All experimental units remained wet regardless the presence or absence of maize plants, by watering the pots with 200 mL of tap water twice a week. Hence, four different sowing dates and a single inoculation time was evaluated, where inoculant bacteria remained on substrate without the plant for up to 8 days. Commercial maize seeds of hybrid 2B610 (Dow AgroSciences) were acquired in the local market and used without any further treatment. Plants were grown for 30 days from the sowing date, and the growth-promotion effects byAb-V5 were determined by biometric parameters: root volume, root dry weight, shoot dry weight and plant dry weight.

#### Field trials

The field trials were conducted in the Fazenda Escola da Universidade Estadual de Londrina (experimental farm of the Londrina State University), at coordinates 23°20′31′′S, 51°12′38′′W, in Londrina, Paraná, Brazil. The experimental site was an Alfisol paleudult (Latossolo Vermelho Eutroférrico, Brazilian Classification) located at an altitude of 540 m, with a climate classified as subtropical (Cfa, according to Köppen's classification). The trials were run in the years 2010/11 and 2012/13, and meteorological data are presented in Supplementary Figure 1. The experimental site was previously cropped with wheat under a no-tillage system in each season, and soil samples from the 0 to 20 cm layer were randomly obtained from 15 points 25 days before the beginning of the experiments for a chemical analysis; the results are presented in Table 2. The maize hybrid AG2040 (Agroceres/Monsanto) was used for the crop season 2010/11, while the maize hybrid 2B587Hx (Dow Agrosciences) was used for the crop season 2012/13.

**Table 2:** Chemical properties of the soil (0–20 cm) in the two seasons (2010/11 and 2012/13) of inoculant trials with maize.

**Crop season**	**pH**	**Al**	**H+Al**	**Ca**	**Mg**	**K**	**P**	**OM^a^**	**CEC^a^**	**BS^a^**
	**H_2_O**	**cmol**_**c**_ **dm**^**−3**^					**mg Kg^−1^**	**g Kg^−1^**	**cmol_c_ Kg^−1^**	**%**
2010/11	5.40	0.01	3.97	6.60	1.30	0.46	10.70	20.6	12.33	67.80
2012/13	5.20	0.11	4.60	5.86	2.35	0.78	10.00	24.1	12.93	69.54

a *OM, soil organic matter; CEC, cation exchange capacity; BS, base saturation*.

Inoculants were prepared from a pre-inoculum of *A. brasilense* Ab-V5 in DYGS liquid media, as stated above, and used to inoculate 250 mL of the culture medium developed to favor high CFU counts and high EPS and PHB concentrations. Bacterial growth was assessed in 1000 mL Erlenmeyer flasks with an initial population of 1 × 10^4^ cells mL^−1^ and an incubation period of 36 h at 28 ± 2°C in a rotary shaker set to 150 rpm. After this period, liquid and peat inoculants were prepared as follows. Liquid inoculants were obtained by diluting the bacterial broth in fresh culture media to a final concentration of 1 × 10^9^ cells mL^−1^, while the peat inoculant was prepared by mixing undiluted bacterial broth into finely ground, sterilized peat to reach a cellular concentration of 1 × 10^9^ cells g^−1^. The liquid and peat inoculants were stored for 30 days in a room with controlled temperature (20 ± 3°C) before being used in the field trials.

The inoculation treatments followed a complete factorial randomized block design with four replications, three N fertilization levels (30, 80, and 160 kg N ha^−1^) and four inoculation treatments (uninoculated control—cont; liquid inoculant over seeds—liq.seed; liquid inoculant as a topdressing—liq.top; and peat inoculant over seeds—peat). The experimental plots comprised six rows 5 m in length, with the working area represented by the four central rows, eliminating 0.5 m at the ends. For each crop season, the soil was prepared prior to sowing by plowing and light harrowing. At the sowing date, the experimental site was fertilized in the sowing furrows with 75 Kg ha^−1^ of P_2_O_5_ as single superphosphate, 130 Kg ha^−1^ of K_2_O as KCl, and 30 Kg ha^−1^of N as urea. In addition, complementary N fertilization was applied at the V_4_ and V_8_ maize development stages to assign the three N fertilization levels studied (30 + 0 + 0 kg N ha^−1^ − 30 kg N ha^−1^; 30 + 25 + 25 kg N ha^−1^ − 80 kg N ha^−1^; and 30 + 75 + 75 kg N ha^−1^ − 160 kg N ha^−1^). Inoculation treatments were performed to reach a population of 3 × 10^6^ *A. brasilense* cells seed^−1^ or plant, according to the type of inoculant tested. To accomplish this, peat inoculant was applied at a dose of 15 mg kg seed^−1^, while liquid inoculant was applied at a dose of 15 mL kg seed^−1^; in both treatments, seed inoculation was performed 12 h before sowing, and the inoculated seeds were stored in a dark, cool and dry place until planting. The topdressing inoculation was achieved by diluting the liquid inoculant formulation in tap water (1:1,000, *v*/*v*), which was applied at a dose of 3 mL per plant (equivalent to 0.003 mL of undiluted inoculant per plant) 10 days after sowing (V_2_ stage) using Costal Spray equipment (Jacto PJH, Pompéia, Brasil) equipped with an air induction nozzle adjusted to 200 μm drops. The efficiency of different *A. brasilense* Ab-V5 inoculation treatments was assessed by examining biometrics, nutritional content and yield, as follows. The stem diameter (mean of 15 plants randomly selected for each replicate) and leaf N content (samples of 20 leaves randomly collected from each experimental plot) were determined at 60 days after planting (VT stage). The stem diameter was obtained using a pachymeter at 20 cm from the ground level; leaf samples were carefully washed in distilled water before being dried at 60°C to a constant weight. The dried leaf samples were then milled and used to determine the total leaf N content by Kjeldahl digestion using a Tecnal TE-0371 digester (Piracicaba, Brazil) to determine the N concentration. In addition, at the end of each crop cycle, the following parameters were recorded: ear size (cm), weight of 100 grains (g), and yield (Kg ha^−1^) at 13% grain humidity.

### Statistics

Statistica software version 7 (Statsoft, Oklahoma) was used for drawing the surface contours, assessing the statistical significance of the regression coefficients (Student's *t*-test) and performing the analysis of variance (ANOVA) to evaluate the statistical significance of the model obtained from the factorial design approach applied to develop the culture medium. All recorded data were tested for distribution normality (Shapiro-Wilk) and variance homogeneity (Bartlett) according to the experimental design. Experimental data were submitted to ANOVA and comparisons of means were conducted using Scott-Knott (greenhouse trial) or Tukey's test (field trials), both with *P* < 0.05. Experimental data from greenhouse and field trials were analyzed using the statistical software R (www.r-project.org) and the ExpDes package. Pearson correlation coefficients were calculated pairwise between the bacterial physiological parameters and the plant growth parameters from greenhouse trial by using the software R and the ggpubr package. The leaf N content was transformed to the root-square scale after the variance homogeneity analysis.

## Results

### Effect of culture media composition on the Ab-V5 biopolymers content

In this study, we assessed the relationships of nutritional components in culture media and the production of EPS and biomass by *A. brasilense* Ab-V5 to define an inoculant formulation and further evaluate its performance in field trials. The relationships between the nutritional components and responses to the dependent variables analyzed are presented in Table 1. Based on these results, a simplified regression equation model for biomass and EPS production was obtained by considering only the significant terms. The regression coefficients calculated from the equations and the ANOVA results are shown in Table 3.

**Table 3:** ANOVA and regression analysis of second-order polynomial model developed according to the results of the surface response methodology for the production of biomass and EPS by *A. brasilense* Ab-V5.

**Factor^a^**	**Biomass**			**EPS**		
	**RC^b^**	***t*-value**	***p*-value**	**RC^b^**	***t*-value**	***p*-value**
β_0_	8.809^**^	57.433	0.000	0.842^**^	11.430	0.000
β_1_	−0.213	−1.275	0.226	−0.200^*^	−2.497	0.028
β_2_	−0.298	−1.784	0.100	0.0824	1.027	0.325
β_4_	0.367^*^	2.195	0.049	0.141	1.760	0.104
β_12_	−0.474^*^	−2.838	0.015	−0.032	−0.401	0.696
β_14_	0.573^**^	3.429	0.005	0.085	1.064	0.308
β_24_	0.558^**^	3.339	0.006	0.426^**^	5.307	0.000
MSR	0.447			0.103		
R^2^	0.772			0.769		
EE^c^	= 8.80895 + (−0.213125 *X*_1_) + (−0.298125 *X*_2_) + (0.366875 *X*_4_) + (−0.474375 *X*_1_*X*_2_) + (0.573125 *X*_1_*X*_4_) + (0.558125 *X*_2_*X*_4_)			= 0.842053 + (−0.200438 *X*_1_) + (0.0824375 *X*_2_) + (0.141312 *X*_4_) + (−0.0321875 *X*_1_*X*_2_) + (0.0854375 *X*_1_*X*_4_) + (0.426063 *X*_2_*X*_4_)		

a *β linear estimates of coefficients for glycerol (β_1_), sucrose (β_2_), K_2_HPO_4_(β_4_) and respective interactions; MSR, mean square residual*.

b *RC, regression coefficient*.

c *EE, expanded equation; X_1_ glycerol concentration (% w/v), X_2_ sucrose concentration (% w/v), X_4_ K_2_HPO_4_ concentration (% w/v)*.

Asterisks denote levels of statistical significance:

* *p < 0.05*,

** *p < 0.01*.

**Figure 1:**
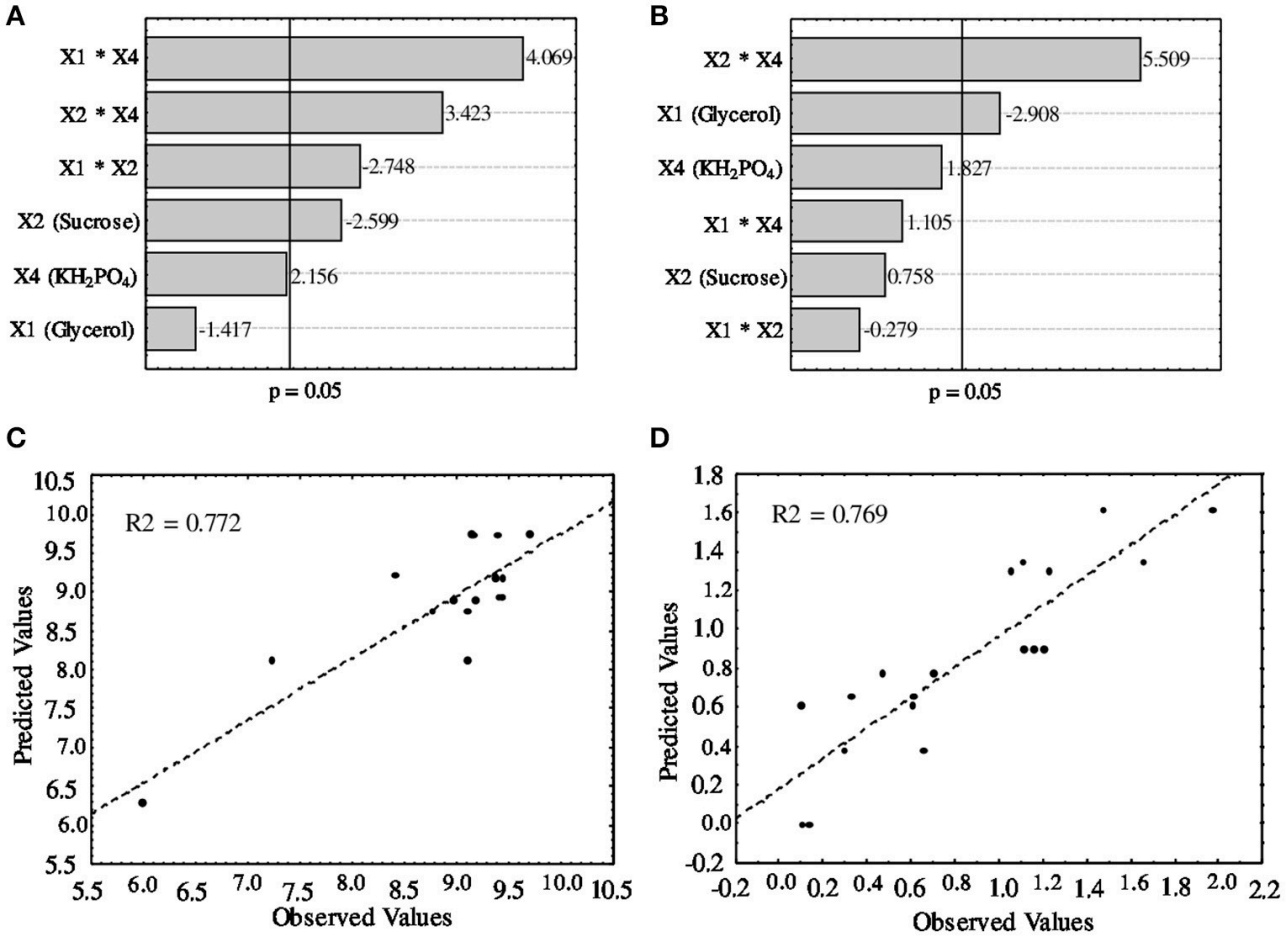
Pareto chart of standardized linear and combined effects estimated from different variables **(A,B)** and parity plots of experimental vs. predicted values **(C,D)** according to the mathematical models developed for *A. brasilense* Ab-V5. **(A)** Pareto chart of estimated effects on biomass productivity; **(B)** Pareto chart of estimated effects on EPS production; **(C)** parity plot of biomass productivity; **(D)** parity plot of EPS production. *X*_1_ glycerol, *X*_2_ sucrose and *X*_4_ K_2_HPO_4_. The line in **(A,B)** indicates the point at which the effect estimates become statistically significant (*p* > 0.05).

As shown by the regression coefficients and the *t* and *p* values, the linear effects of the independent variables were related to a positive effect of potassium monohydrogen phosphate on the biomass productivity of *A. brasilense*, as well as a negative effect of glycerol on EPS production. In addition, although the effects of both glycerol and sucrose were not statistically significant, their linear or combined effects negatively influenced the biomass productivity; these negative effects were overcome when glycerol or sucrose interacted with KH_2_PO_4_, by assuming a positive interaction effect. Glycerol also had a negative influence on the response of both dependent variables studied, while sucrose showed a negative regression coefficient value for biomass productivity and a positive value for EPS production (Figures 1A,B). The potassium monohydrogen phosphate positively influenced both dependent variables studied, even considering its combined effect with glycerol or sucrose. The second-order polynomial mathematical model constructed from the experimental data indicates a linear relation (Figures 1C,D) between the predicted and observed values for the dependent variables, with R^2^ values of 0.772 and 0.769 for biomass productivity and EPS production, respectively. These results also indicate that ~23% of the observed responses could not be explained by the model, suggesting that further improvements could be achieved by modifying the independent variables.

**Figure 2:**
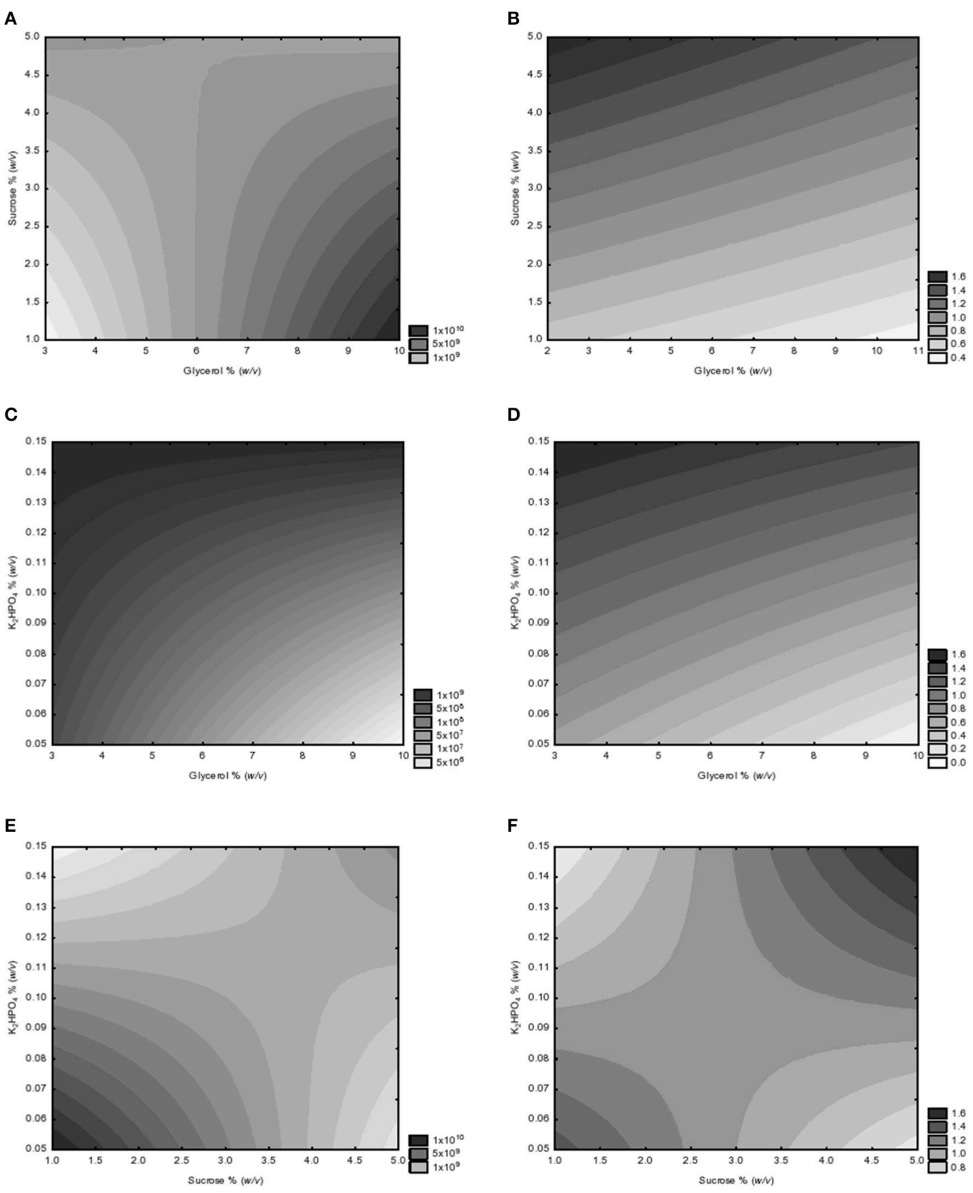
Response surface plots (RSPs) of the combined effects of independent variables (X_1_ vs. X_2_, X_1_ vs. X_4_, and X_2_ vs. X_4_) on the biomass productivity **(A,C,D)** and EPS production **(B,D,E)** of *A. brasilense* Ab-V5. **(A)** RSP of the combined effects of glycerol and sucrose on biomass productivity (CFU counts); **(B)** RSP of the combined effects of glycerol and sucrose on EPS production; **(C)** RSP of the combined effects of glycerol and K_2_HPO_4_ on biomass productivity; **(D)** RSP of the combined effects of glycerol and K2HPO4 on EPS production; **(E)** RSP of the combined effects of sucrose and K_2_HPO_4_ on biomass productivity; **(F)** RSP of the combined effects of sucrose and K_2_HPO_4_ on EPS production. Expanded equations related to the CFU and EPS plots are presented in Table 3.

The response surface plots defined by the regression models are presented in the Figure 2, where the yeast extract was kept constant (1% *w*/*v*) in all evaluations due to the absence of its effect over the independent variables studied. It is interesting to observe that the effect of the independent variables glycerol (*X*_1_) and sucrose (*X*_2_) on biomass productivity or EPS production were divergent when the KH_2_PO_4_ concentration (*X*_4_) was kept constant (0.15%, *w*/*v*). While maximum CFU counts was observed at the highest glycerol and the lowest sucrose concentrations (10 and 1% *w*/*v*, respectively; Figure 2A), maximum EPS production was observed at the lowest glycerol (3% *w*/*v*) and the highest sucrose (5% *w*/*v*) levels (Figure 2B). This same pattern was observed when plotting the independent variables glycerol (*X*_1_) and K_2_HPO_4_ (*X*_4_) with a constant sucrose concentration of 5% (*w*/*v*), resulting in maximum CFU counts at the highest glycerol and K_2_HPO_4_ (0.15% *w*/*v*) levels (Figure 2C), while maximum EPS occurred at the lowest glycerol and K_2_HPO_4_ (0.05% *w*/*v*) levels (Figure 2D). The combined effect of sucrose (*X*_2_) and K_2_HPO_4_ (*X*_4_) at a constant glycerol concentration (3% *w*/*v*) confirmed the apparent inhibitory effect of sucrose on CFU counts (Figure 2E), meanwhile stimulating the EPS production (Figure 2F).

According to the experimental results, the concentration of materials used in run 15 (glycerol 3% *w/v*; sucrose 5% *w/v*; yeast extract 5% *w/v*; K_2_HPO_4_ 0.15% *w/v*) resulted in the best composition (desirability of 0.79, Supplementary Figure 2) for yielding cultures of *A. brasilense* Ab-V5 with a high population density (up to 2.34 × 10^9^ cells mL^−1^) and a high EPS content (up to 1.97 g L^−1^). As such, this formulation was selected for further studies and is referred to as Form15 in this manuscript.

### *Azospirillum brasilense* Ab-V5 growth kinetics and cellular status in the culture medium Form15

**Figure 3:**
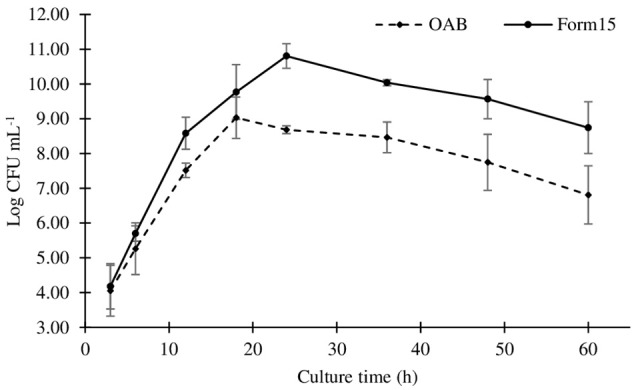
Growth kinetics of *Azospirillum brasilense* Ab-V5 measured as colony forming units in the culture medium OAB (Bashan and de-Bashan, 2015) and in Form15 (this work); 250 mL shaker flasks filled with 50 mL of culture medium were maintained at 28 ± 2°C and 150 rpm shaking frequency.

The comparative growth kinetics of *A. brasilense* Ab-V5 in Form15 and in the OAB culture medium are presented in Figure 3. Is possible to observe that bacterial growth was improved using Form15, and the kinetic curves started to diverge as soon as after 6 h of culture. Form15 also extended the log phase of growth by ~6 h and delayed the population decay of *A. brasilense*, while in the OAB culture medium, the end of the log phase took place after 18 h of cultivation. In addition, the maximum CFU counts in Form15 were 2.25 × 10^10^ cells mL^−1^ (24 h of growth), while those of the OAB medium were 1.07 × 10^9^ cells mL^−1^ (18 h of growth), illustrating an enhancing effect of 21.03-fold.

The bacterium *A. brasilense* differed greatly in relation to its physiological status according to the culture medium used for its cultivation. Table 4 presents the levels of EPS and PHB production, as well as the biomass accumulation resulting from *A. brasilense* growth in both culture media studied. Form15 better supported *A. brasilense* growth, resulting in higher biomass accumulation, which agreed with the kinetics data. Form15 resulted in a more rapid increase in *A. brasilense* biomass than did the OAB medium, with a short generation time and a higher growth rate. Consequently, the amount of cell biomass that accumulated in the OAB medium after 60 h of growth was half of the biomass that accumulated in Form15 after just 12 h of growth. The nutritional composition of Form 15 promoted a huge increase (ranging from 1,654 to 1,805%) in the EPS production of *A. brasilense* compared with the OAB medium, where the EPS concentration did not exhibit significant increases during the experiment. On the other hand, although the amount of PHB recovered from *A. brasilense* cultures was greater when grown in Form15 (due to the higher biomass production), the ratio of the amount of intracellular PHB and the biomass of *A. brasilense* cultivated in Form15 was about half of the value observed for the OAB culture medium. In addition, *A. brasilense* cells accumulated PHB at a faster rate when grown in OAB than in Form15, with PHB accounting for 33.3 and 16.7% of the cell biomass weight of the cultures grown in the OAB and Form15 respectively, after 36 h of culture.

**Table 4:** Growth rate (μ), generation time (*g*), biomass production, EPS concentration, PHB content, and EPS:biomass and PHB:biomass ratios of *Azospirillum brasilense* Ab-V5 grown in different culture media and evaluated at different periods of culturing in the culture medium OAB (Bashan and de-Bashan, 2015) and in Form15 (this work).

**Parameters**	**Culture time (h)**					
	**12**		**36**		**60**	
	**OAB^a^**	**Form15^a^**	**OAB**	**Form15**	**OAB**	**Form15**
μ (h^−1^)	0.68 ± 0.06 b	0.88 ± 0.02 a	0.29 ± 0.003 d	0.39 ± 0.003 c	0.11 ± 0.002 e	0.18 ± 0.005 e
*g* (h)	1.09 ± 0.09 e	0.82 ± 0.03 f	1.95 ± 0.01 c	1.65 ± 0.01 d	2.92 ± 0.02 a	2.40 ± 0.03 b
Biomass (g L^−1^)	0.19 ± 0.03 e	1.11 ± 0.10 c	0.37 ± 0.13 de	2.05 ± 0.01 b	0.55 ± 0.07 d	2.37 ± 0.05 a
EPS (g L^−1^)	0.13 ± 0.08 c	2.15 ± 0.52 b	0.18 ± 0.18 c	2.93 ± 0.49 b	0.22 ± 0.30 c	3.97 ± 1.30 a
PHB (mg L^−1^)	1.35 ± 0.02 e	6.09 ± 0.22 e	122.24 ± 1.43 d	341.60 ± 2.62 b	244.34 ± 1.12 c	455.92 ± 4.60 a
EPS:biomass ratio (g:g)	0.70 ± 0.28 b	1.93 ± 0.33 a	0.48 ± 0.11 b	1.43 ± 0.15 a	0.41 ± 0.07 b	1.68 ± 0.38 a
PHB:biomass ratio (mg:g)	7.04 ± 1.01 d	5.48 ± 1.35 d	332.79 ± 35.61 b	166.80 ± 7.02 c	445.67 ± 37.18 a	192.37 ± 15.33 c

a *OAB: modified NFb culture medium (Bashan and de-Bashan, 2015); Form15: culture medium described in this work. Bacterial cultures were grown in 1000 mL shaker flasks filled with 250 mL of culture medium and maintained at 28 ± 2°C and 150 rpm shaking frequency. Significant differences (P < 0.05) according to Tukey's test are indicated by small letters when comparing contrasts in rows*.

### Performance of *A. brasilense* Ab-V5 grown in Form15 as maize inoculant

#### Pot trial

**Figure 4:**
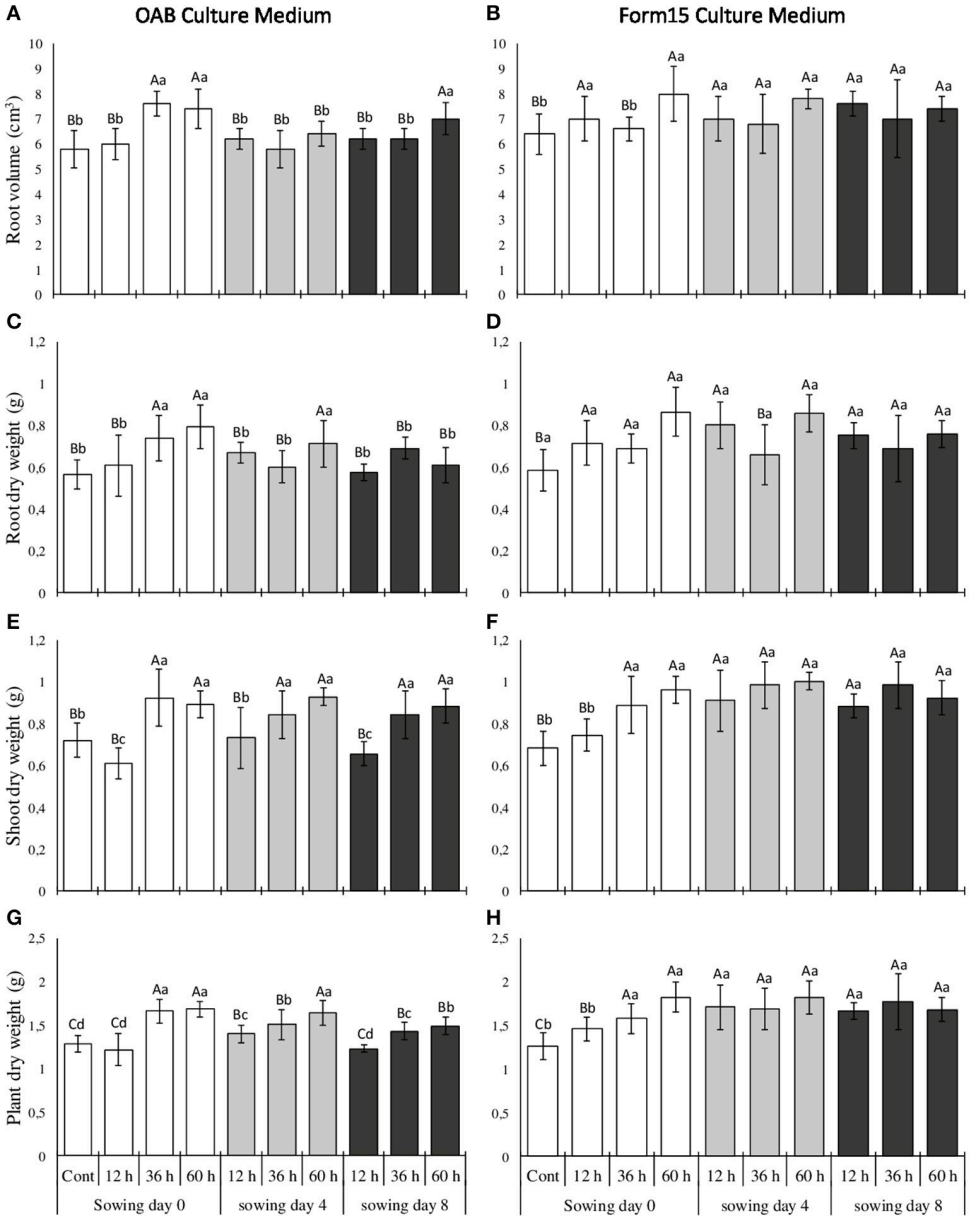
Root volume **(A,B)**, root dry weight **(C,D)**, shoot dry weight **(E,F)** and plant dry weight **(G,H)** of maize plants grown in pots filled with a mixture of sand and oxisol in a proportion of 2:1 (*v*:*v*) as substrate. Substrate were inoculated with *Azospirillum brasilense* inoculants prepared from cultures of 12, 36, and 60 h of growth in OAB **(A,C,E,G)** or Form15 **(B,D,F,H)** culture medium. Inoculants were set down 2–3 cm below the substrate surface, and maize seeds were planted on different dates (0, 4, and 8 days) after substrate inoculation. Significant differences (*p* < 0.05) according to the Skott-Knott test are shown in lowercase letters for each culture medium or in capital letters between the culture media. Control treatments are related to substrate inoculated with sterile OAB **(A,C,E,G)** or Form15 **(B,D,F,H)** culture media.

One-way analysis of variance (ANOVA) and the means comparison of growth-promotion effects induced by *A. brasilense* Ab-V5 were performed for each culture media (OAB and Form15) separately (small letters) and combined (capital letters), and are presented in Figure 4 respectively. The results demonstrate that an increase in cultivation time of inoculant cells, which is related to the increase in the PHB and EPS cellular contents (Table 4), resulted in higher growth-promotion responses in maize when seeds were sown just after the substrate inoculation with Ab-V5 (sowing day 0, white bars). At day 0 sowing time, the Ab-V5 inocula prepared from their cultivation in any culture media promoted significant differences in the maize growth as compared to control (hatched bar), but only when the cultures lasted at least 36 h. An exception was the root volume that increased in plants sown in pots inoculated with Ab-V5 prepared from cultivation in the Form15 regardless the culturing time last. At day 0, the combined analysis of maize growth in response to Ab-V5 association (capital letters) reinforces the interrelationship between the time that inocula were cultured and the growth-promotion effects, but also indicates that inocula prepared from Form15 had a superior performance than those from OAB culture medium. This can be noted by the contrasts in the root volume, root dry weight and plant dry weight of maize grown in pots inoculated with 12-h cultured inocula.

At later sowing times, the response of maize to Ab-V5 varied according to the culture media used to prepare the inocula and the elapsed time between substrate inoculation and planting. Separate analysis of growth-promotion induced by Ab-V5 cultured in OAB or in Form15 media and inoculated in the substrate 4 days before maize sowing (light gray bars, small letters) indicates that bacteria viability was lowered in OAB-derived inocula when compared to Form15-derived inocula. Whilst the root volume of plants grown in pots inoculated with OAB inocula showed no differences to the control plants, regardless the culture period of the inoculant bacteria lasts (12, 36, or 60 h of culturing), the root volume of plants grown in pots inoculated with Form15 inocula showed the opposite result. Similar effect was noted for the root dry weight, although plants grown in pots inoculated with Ab-V5 grown for 60 h in OAB medium had increased the root dry weight in relation to control plants, and those plants grown in pots added with Form15 inoculum from 36-h cultures had no increase in the root dry weight. In addition, the dry weight of maize shoots was increased for all inocula except for the plants grown in pots that received 12-h OAB inoculum. The plant dry weight was increased in maize grown with substrate added with OAB inocula from any culture time, although the growth-promotion effect had increased concomitantly as the culture time increased. On the other hand, plants grown in pots inoculated with Ab-V5 from Form15 showed higher values of dry weight than control plants disregarding the culture time that the inocula lasts. Combined analysis of plant growth-promotion at day 4 sowing time (capital letters) confirms that maize plants performed better when grown in pots inoculated with Form15 inocula than plants grown in pots inoculated with OAB inocula, and suggests that compared to the OAB, culture medium Form15 results in higher viability to Ab-V5 cells when introduced to the soil.

The latter seeding time studied was 8 days after the substrate inoculation, where the plant response to the association with *A. brasilense* became clearly distinct between OAB and Form15-derived inocula (dark gray bars). For Ab-V5 inocula prepared from OAB, the effectiveness of growth promotion in response to the association with this bacterium was restrict to few treatment combinations, and were always related to long-time cultures. On the opposite hand, the inocula prepared from Form15 supported high values for all the parameters evaluated, regardless the time of culturing adopted to prepare the inocula. The combined evaluation of plant growth at the latter sowing date (capital letters) reinforces the increased viability of Ab-V5 cultured in Form15 as compared to the OAB culture medium, due to the comparatively higher values observed in all the growth parameters studied that did not differ from the maximum values recorded. To further explore significant dependencies between physiological parameters of *A. brasilense* Ab-V5 and plant response to its inoculation at different culturing times, a correlation analysis between the biopolymers produced under different bacterial growth conditions with the plant biometric parameters was carried out, and the resulting strength and direction of associations are presented in Table 5. According to these results, EPS concentration of Ab-V5 biomass from the OAB medium was negatively correlated whereas the PHB content was positively correlated with maize growth, except for the root dry weight. Controversially, when Ab-V5 was cultured in Form15 no correlation between EPS and the plant growth was found significant, while the PHB content had a single correlation with the shoot dry weight. A combined correlation analysis considering the values from both culture media resulted in a positive correlation between the EPS and all the plant growth parameters, although the PHB content has only been correlated with the shoot dry weight. The EPS concentration and PHB content of Ab-V5 cells had negative correlation each other, regardless the culture media used to produce the bacterial biomass.

**Table 5:** Correlation analysis between the EPS concentration and PHB content of Ab-V5 biomass from different culture media and the biometric parameters used to determine the maize growth.

**Correlation source^a^**	**Culture media**	**Df^b^**	**RC^c^**	***t*-value**	***p*-value**
EPS × Root volume	OAB	43	−0.374^**^	−2.648	0.011
EPS × Root dry weight	OAB	43	−0.280	−1.914	0.062
EPS × Shoot dry weight	OAB	43	−0.815^**^	−9.230	9.282e-12
EPS × Plant dry weight	OAB	43	−0.676^**^	−6.018	3.447e-07
EPS × Root volume	Form15	43	0.173	1.155	0.255
EPS × Root dry weight	Form15	43	0.252	1.706	0.095
EPS × Shoot dry weight	Form15	43	−0.280	−1.910	0.063
EPS × Plant dry weight	Form15	43	−0.118	−0.779	0.440
EPS × Root volume	OAB + Form15	88	0.333^**^	3.312	0.001
EPS × Root dry weight	OAB + Form15	88	0.331^**^	3.293	0.001
EPS × Shoot dry weight	OAB + Form15	88	0.209^*^	2.000	0.049
EPS × Plant dry weight	OAB + Form15	88	0.340^**^	3.388	0.001
PHB × Root volume	OAB	43	0.376^**^	2.660	0.012
PHB × Root dry weight	OAB	43	0.283	1.934	0.060
PHB × Shoot dry weight	OAB	43	0.812^**^	9.132	1.261e-11
PHB × Plant dry weight	OAB	43	0.676^**^	6.012	3.508e-07
PHB × Root volume	Form15	43	0.003	0.022	0.983
PHB × Root dry weight	Form15	43	−0.046	−0.300	0.765
PHB × Shoot dry weight	Form15	43	0.398^**^	2.844	0.007
PHB × Plant dry weight	Form15	43	0.247	1.671	0.102
PHB × Root volume	OAB + Form15	88	0.032	0.301	0.764
PHB × Root dry weight	OAB + Form15	88	−0.004	−0.038	0.97
PHB × Shoot dry weight	OAB + Form15	88	0.400^**^	4.102	9.126e-05
PHB × Plant dry weight	OAB + Form15	88	0.198	1.895	0.061
PHB × EPS	OAB	7	−0.565	−1.814	0.113
PHB × EPS	Form15	7	−0.442	−1.303	0.234
PHB × EPS	OAB + Form15	16	−0.551^**^	−2.614	0.018

a *Correlations were performed between the EPS:biomass and PHB:biomass values from each or combined culture media and the values of plant growth for 30 days under greenhouse conditions*.

b *Df degrees of freedom*.

c *RC regression coefficient*.

Asterisks denote levels of statistical significance:

* *p < 0.05*,

** *p < 0.01*.

#### Field trials

*Azospirillum brasilense* Ab-V5 liquid and peat-based inoculants prepared using the Form15 culture medium were tested in two different crop seasons, under different N fertilizer doses. Table 6 presents the ANOVA data obtained from both crop seasons, considering the effects of the inoculation treatments, N fertilizer doses and respective interactions with the stem diameter, total nitrogen content on leaves, ear length, number of grains per ear, mass of 100 grains and yield. Considering the 2010/11 crop season (maize genotype AG2040), the ANOVA showed an effect of N fertilizer doses on the stem diameter (SAD), while the inoculation treatments influenced the leaf N concentration (NLC), ear length (EAL), the weight and number of grains per ear (W100 and NG, respectively), and grain yield. In this same sense, the ANOVA for the 2012/13 crop season (maize genotype 2B587Hx) demonstrates that the N fertilizer influenced the stem diameter, leaf N content and weight of grains; moreover, the inoculation treatments influenced the N leaf content and yield.

**Table 6:** ANOVA mean square values for productivity and nutritional parameters in two maize varieties grown in different crop seasons in response to different N fertilization levels and different inoculants formulated with *A. brasilense* Ab-V5.

**Effects**	**df**	**Mean Square**^**a**^					
		**SAD^b^**	**NLC**	**EAL**	**W100**	**NG**	**YIELD**
**MAIZE GENOTYPE AG2040 (MONSANTO)–2010/11 CROP SEASON**							
N fertilizer level (*N*)^c^	2	2.96^*^	4.41	1.69	14.08	656.96	1080191
Inoculant treatments (*I*)^d^	3	0.89	54.23^**^	1.70^*^	36.08^**^	3, 163.24^**^	6534672^**^
*N* × *I*	6	0.31	17.71^**^	0.92	7.08	1, 380.09^**^	2716462^*^
CV (%)		5.31	25.62	5.37	7.95	4.32	12.47
Error	33	1.03	3.60	0.56	7.32	417.69	1128940
*I* × 30 Kg N ha^−1^	3	0.42	26.58^**^	2.25^*^	38.92^**^	3, 621.17^**^	9407003^**^
*I* × 80 Kg N ha^−1^	3	0.98	8.48	1.18	8.67	1, 496.22^**^	2541662^**^
*I* × 160 Kg N ha^−1^	3	0.11	26.94^**^	0.12	2.67	806.04	425076
*N* × cont	2	1.39	8.54	2.56^*^	19.00^*^	1, 465.04^*^	6451233^**^
*N* × peat	2	0.65	0.81	0.06	4.00	1, 928.01	813308
*N* × liq.seed	2	0.20	5.92	0.09	3.00	1, 351.96^*^	683055
*N* × liq.top	2	1.65	0.82	1.75	9.33	52.23	3035959^*^
**MAIZE GENOTYPE 2B587HX (DOW AGROSCIENCES)–2012/13 CROP SEASON**							
N fertilizer level (*N*)	2	1.91^*^	35.44^*^	1.12	4.55^*^	197.35	701164
Inoculant treatment (*I*)	3	0.49	97.22^*^	1.59	0.27	251.35	842100^*^
*N* × *I*	6	0.92	14.69	0.34	1.68	1, 085.97^*^	423408
CV (%)		4.62	19.44	5.93	2.94	3.07	5.13
Error	33	0.53	8.21	0.73	0.99	343.52	270610
*I* × 30 Kg N ha^−1^	3	1.83^*^	84.33^**^	1.81	1.24	1, 541.02^*^	1393969^**^
*I* × 80 Kg N ha^−1^	3	0.37	12.93	0.39	1.29	133.60	193568
*I* × 160 Kg N ha^−1^	3	0.14	29.33^*^	0.06	1.10	748.70	101377
*N* × cont	2	0.65	83.21^**^	1.55	0.33	1, 825.99	1536779^**^
*N* × peat	2	3.09	3.77	0.36	4.26^*^	469.42	158073
*N* × liq.seed	2	0.51	32.39^*^	0.08	4.92^*^	335.19	154756
*N* × liq.top	2	0.42	10.85	0.14	0.09	824.70^*^	121777

a Asterisks denote levels of statistical significance: ^*^P < 0.05, ^**^P < 0.01.

b *SAD, stem diameter; NLC, leaf N content; EAL, ear length; W100, weight of 100 grains; NG, number of grains per ear; YIELD, grain productivity. NLC, data were transformed by $\sqrt{x}$*.

c *N fertilizer levels: 30, 80, and 160 Kg N ha^−1^*.

d *Inoculant treatments: cont, uninoculated control; peat, peat inoculant over seeds; liq.seed, liquid inoculant over seeds: liq.top, liquid inoculant as a topdressing*.

The interaction effect between the N fertilizer and the inoculation treatments was significant only for the AG2040 genotype, influencing the leaf N content and grain yield. Although the interaction effect appeared to be negligible for the series of variables studied, when each N fertilizer and inoculation treatment was considered, we could identify significant effects that were diluted in the mean value of all interactions. This approach highlighted the non-additive effect between the inoculation treatments and the use of higher N fertilizer doses, as observed by the inoculation effects at low N fertilizer input, as well as by the absence of an N fertilization effect in the treatments where the inoculation was performed, regardless of the variety considered. This non-additive effect is better noted when comparing the treatment means presented in Table 7, no significant effects on the parameters studied were observed in maize plants that received 160 kg N ha^−1^ in addition to an *A. brasilense* inoculation (independently of the inoculation method), except for the N leaf content in both maize varieties. In contrast, maize plants grown under the lowest N fertilization dose (30 kg N ha^−1^) presented increases in all parameters studied except for the stem diameter in the AG2040 variety and ear length and grain weight in the 2B587Hx variety. Grain yield responded to N fertilizer only in uninoculated plants, while inoculation with *A. brasilense* increased the maize yield at lower N fertilizer doses to a productivity level equal to that achieved with higher N fertilization doses. The yield increased for both varieties in response to inoculation with *A. brasilense* Ab-V5, especially when using the peat-based inoculant, although the use of the liquid inoculant formulation before planting (i.e., the seed treatment) or as a topdressing indeed showed productivity levels comparable to those obtained with the use of higher N fertilization doses.

**Table 7:** Mean values for productivity and nutritional parameters in two maize varieties grown in different crop seasons under different N fertilization levels and different inoculant treatments with *A. brasilense* Ab-V5.

**Trait^a^**	**N Level^b^**	**Maize genotype AG2040**					**Maize genotype 2B587Hx**				
		**Inoculant treatments**^**c**^					**Inoculant treatments**^**c**^				
		**Cont**	**Peat**	**Liq.seed**	**Liq.post**	**N Level effect**	**Cont**	**Peat**	**Liq.seed**	**Liq.post**	**N Level effect**
SAD	30 kg ha^−1^	13.6	14.0	14.3	13.7	13.9 B	15.1 ab	14.6 b	16.2 a	15.5 ab	15.3 B
	80 kg ha^−1^	13.7	14.7	14.7	14.5	14.4 AB	15.8	16.2	15.5	16.0	15.9 AB
	160 kg ha^−1^	14.6	14.7	14.7	15.0	14.8 A	15.7	16.0	16.1	16.2	16,0 A
Inoculation effect	14.0	14.5	14.6	14.4		15.5	15.6	15.9	15.9	
NLC	30 kg ha^−1^	9.42 b	13.08 ab	20.41 a	12.13 b	13.76	15.96 Cc	23.41 b	29.71 Aa	25.36 ab	23.61 B
	80 kg ha^−1^	11.17	15.60	17.47	13.56	14.45	23.06 B	21.46	25.29 B	23.06	23.22 B
	160 kg ha^−1^	16.92 ab	17.51 b	19.20 a	17.08 b	17.68	29.37 Aab	27.77 b	31.20 Aa	24.44 ab	28.19 A
Inoculation effect	12.51 b	15.40 b	19.02 a	14.26 a		25.28 ab	21.73 b	28.73 a	24.29 ab	
EAL	30 kg ha^−1^	12.9 Bb	14.6 a	14.2 ab	13.3 ab	13.7	13.3	14.1	14.3	14.9	14.2
	80 kg ha^−1^	13.3 AB	14.5	14.4	14.3	14.1	14.3	14.7	14.6	15.0	14.7
	160 kg ha^−1^	14.5 A	14.3	14.2	14.6	14.4	14.4	14.6	14.6	14.7	14.6
Inoculation effect	13.6 b	14.4 a	14.3 ab	14.1 ab		14.0	14.5	14.5	14.9	
NG	30 kg ha^−1^	383.7 Bb	445.3 a	446.4 Aa	438.7 ab	428.5	578.5 b	607.2 ab	610.9 ab	625.4 Aa	605.5
	80 kg ha^−1^	409.3 Ac	451.8 a	426.1 Bbc	445.6 ab	433.2	604.6	613.2	600.7	611.1 AB	607.4
	160 kg ha^−1^	421.1 A	410.9	409.7 B	440.4	420.5	620.9	592.2	592.6	596.7 B	600.3
Inoculation effect	404.7 b	436.0 a	427.4 ab	441.6 a		601.3	604.2	601.4	611.1	
W100	30 kg ha^−1^	30.1 Bb	35.4 ab	36.5 a	31.2 b	33.3	33.4	32.5 B	33.2 B	33.9	33.3 B
	80 kg ha^−1^	35.2 A	36.3	38.1	34.3	36.0	33.9	34.1 AB	33.0 B	34.2	33.8 AB
	160 kg ha^−1^	34.1 A	34.4	35.7	33.2	34.3	33.8	34.5 A	35.0 A	34.0	34.3 A
Inoculation effect	33.1 b	35.4 ab	36.8 a	32.9 b		33.7	33.7	33.7	34.0	
YIELD	30 kg ha^−1^	6,353.6 Cc	9,746.9 a	8,863.0 ab	7,528.0 Bbc	8,122.9	9,349.4 Bb	10,325.0 a	9,905.5 ab	10,470.9 a	10,012.7
	80 kg ha^−1^	7,466.3 Bb	8,978.6 a	8,294.2 ab	9,252.6 Aa	8,497	10,140.8 A	10,271.7	9,972.9	10,269.1	10,163.6
	160 kg ha^−1^	8,775.7 A	8,871.5	8,820.7	8,175.4 B	8,660.8	10,164.8 A	10,510.1	10,274.9	10,181.0	10,282.7
Inoculation effect	7,531.9 b	9,199.0 a	8,659.3 a	8,318.3 ab		9,885.0 b	10,369.0 a	10,051.1 ab	10,307.0 ab	

*Differences between means were determined by Tukey's HSD test. Significant differences (p < 0.05) according to Tukey's test are indicated by different capital letters when comparing contrasts in columns and different small letters when comparing contrasts in rows, and not significant differences are indicated by omitting notation letters*.

a *SAD, stem diameter (mm); NLC, leaf N content (g kg^−1^); EAL, ear length (cm); NG, number of grains per ear; W100, weight of 100 grains (g); YIELD, grain productivity (kg ha^−1^). NLC, data were transformed by $\sqrt{x}$*.

b *N fertilizer levels: 30, 80, and 160 Kg N ha^−1^*.

c *Inoculant treatments: cont, uninoculated control; peat, peat inoculant over seeds; liq.seed, liquid inoculant over seeds; liq.top, liquid inoculant at topdressing*.

## Discussion

Different *Azospirillum* species were reported to positively influence the development and productivity of a wide variety of plant species, being by far the most studied PGPB group and considered a model for studies in plant-bacteria interactions (de-Bashan et al., 2016). It has also been stated that to promote the growth of a particular plant species, there is a need for Azospirilla build up high populations in the rhizosphere to efficiently colonize the root system. In doing this, the inoculated bacteria can surpass the native soil microbial communities in the competition for the resources made available through root exudates, leading to proper establishment and cooperation with the host plant, as well as the efficient expression of the intended beneficial effect (Bashan et al., 2014; Pereg et al., 2016). To reach this objective and expand the use of PGPB inoculants as a routine agricultural practice able to sustain high crop yields under low or nule amounts of chemical fertilizers or pesticides inputs, the intended benefits expected from the inoculation must be less subjected to variability in their effectiveness and less dependent on favorable environmental factors.

Based on a surface response approach, the development of a culture medium for *A. brasilense* Ab-V5 that promotes a high population density with high amounts of EPS and PHB was addressed. Culture media composition has major influence on the growth and physiological status of a given bacteria, mainly considering responses related to the secondary metabolism which are associated with important ecological functions such as competition, intra and intercellular communication and survival (Karlovsky, 2008). Hence, optimized production for a given metabolite involves the modification of the microbial growth conditions which can be achieved through different methods. Among the most adopted strategies, the response surface methodology has advantages because it allows the standardization of several components at same time and quantifies the impact of each component, as single or interactive effect with the other components, over the productivity of the target response (Maddox and Richert, 1977; Managamuri et al., 2016). The development of culture media for the mass production of *A. brasilense* has been previously addressed, but few reports have presented the physiological conditions of the bacteria after the growth period. The main goals of such studies are to develop culture media using low-cost materials suitable for industrial use and producing high bacterial population densities of the inoculant strain (Bashan et al., 2011; Trujillo-Roldán et al., 2013; Bashan and de-Bashan, 2015). For instance, Bashan et al. (2011) increased the growth rate and the final *A. brasilense* population from 114 to up to 232% by changing the carbon source in the culture medium from glucose to glycerol or gluconate, respectively. Dissolved oxygen also influences the growth of *A. brasilense*; an increased lag phase and decreased specific growth rate were observed at dissolved oxygen tensions (DOTs) higher than 85%, while lower DOTs (~3%) increased the growth rate (Trujillo-Roldán et al., 2013) and yielded a high accumulation of polyhydroxybutyrate (Carrasco-Espinosa et al., 2015).

In our study, although the main goal was not to obtain a low-cost culture medium, the four substances used as the main components can be easily found at low cost on the global market. Glycerol and sucrose are the main carbon sources in the culture media developed, in addition to the relatively high amounts of yeast extract used as a source of growth factors. Glycerol has interesting characteristics which favors its use in inoculant formulations: it can be converted by few steps into an intermediate of glycolysis and used as a carbon source for biomass production, as well as function as protective agent for the microbial cells against desiccation due to its osmoprotectant characteristic (Vassilev et al., 2017). The substitution of glucose by glycerol in culture media used for *Azospirillum* growth has been previously demonstrated to increase the growth rate and decrease the generation time, while producing high bacterial population densities (Bashan et al., 2011), although its effect on the EPS production has not yet been addressed. Sucrose is used as addictive in liquid inoculant formulations of rhizobia and other PGPB because its role in the improvement of bacterial survival; it is also used as adhesive when coating seeds with peat-based inoculants is performed (Bashan et al., 2014). On the other hand, *A. brasilense* is not considered capable of using sucrose as carbon source (Hartmann and Zimmer, 1994; Sahoo et al., 2014), although the genes coding for the enzymes related to the metabolism of glucose and fructose are present in their genome (Wisniewski-Dyé et al., 2011). The importance of sucrose for the production of biomass and EPS by *A. brasilense* Ab-V5 was demonstrated in the present study, although its concentration influenced the CFU counts and EPS production only when considering the combined effect, which was negative when together with glycerol and positive with K_2_HPO_4_. Even considering that Ab-V5 had preferentially used glycerol in the selected formulation, the EPS content was negatively impacted by its content while sucrose concentration had improved the EPS production as observed on the response surface plots. Although further investigation is needed, the spontaneous hydrolysis of sucrose (Wolfenden and Yuan, 2008) is an achievable event along the bacterial culturing and may have made available to *A. brasilense* its constituent monosaccharides to build up the EPS, once glucose comprises as much as half of the sugars present in the extracellular matrix (Fischer et al., 2003).

The nitrogen concentration in the culture media influences important physiological characteristics in *Azospirillum*, such as vitamin production, cell shape, structure of capsular polysaccharides and phytormones production (Palacios et al., 2014; Cassán et al., 2015; Yevstigneyeva et al., 2016), in addition to the synthesis of EPS and PHB (Okon and Itzigsohn, 1992; Sun et al., 2000; Tsagou et al., 2003). Is important to note that Form15 contains a relatively high amount of yeast extract (YE) for the N supply, and the concentrations of glycerol, sucrose and yeast extract were unusually high in this formulation compared with those in other culture media normally adopted to produce PGPB cellular biomass. Nevertheless, no significant effects of YE on the dependent variables were identified by the factorial design, and a comparative analysis of the formulation used in run 11 (Form11) with Form15—which differed only in the amount of YE, indicated that the former exhibited a lower bacterial population density and EPS content. The regression model confirmed this result by indicating the concentrations of independent variables at the values found in Form15, including the YE concentration at the maximum value (Supplementary Figure 2). Although we did not use the regression model to optimize the culture medium composition (i.e., concentrations of glycerol, sucrose, yeast extract and KH_2_PO_4_) in this study, the profiles of the predicted values and desirability indicated that increased CFU counts and EPS production could be obtained by using higher concentrations of sucrose and KH_2_PO_4_ (Supplementary Figure 2). The intended effect of adding YE to culture media, usually at a concentration of 0.1–0.5%, is to stimulate bacterial growth by supplying organic nutrients. Despite this, its concentration has been shown to influence the production of EPS by *Xanthomonas campestris* (Lo et al., 1997), and the results from the factorial design approach indicate that the combined effect of YE with the other independent variables has a similar outcome in *A. brasilense* Ab-V5 EPS production. Additionally, although the intracellular PHB content was not considered in selecting the culture medium suitable for producing *A. brasilense* cells, the bacterial growth in Form15 resulted in an PHB accumulation of ~16.7% of cell dry mass after 36 h of culture.

Altogether, the combination of glycerol, sucrose and YE in the concentrations defined in Form15 have supported the growth of high *A. brasilense* populations and resulted in bacterial cultures enriched in EPS and intracellular PHB. Based on previous studies (van Veen et al., 1997; Catroux et al., 2001; Bashan et al., 2014), these characteristics were adopted in this work as parameters for defining the physiological status of *Azospirillum* with the aim of producing high-performance inoculants. The metabolisms of PHB and EPS have been shown to be induced in response to environmentally stressful conditions and appear to be interrelated in *A. brasilense* and other bacterial genera, where mutants impaired in PHB production demonstrate increases in EPS production (Kadouri et al., 2003; Rodriguez et al., 2006; Cassán et al., 2015). Thus, Form15 can be considered an innovation in cellular biomass production for use in agricultural inoculants as it leads to high CFU counts for *A. brasilense* Ab-V5, which could indeed produce EPS and PHB in high amounts under non-limiting nutritional conditions. It is noteworthy that *A. brasilense* Ab-V5 cultured in Form15 showed an extended log phase, a higher growth rate and a shorter generation time than the OAB medium. Although the shelf-life of inoculants prepared from cells grown in Form15 has not been addressed in this study, the effectiveness of this formulation in extend the viability of Ab-V5 cells in soil was confirmed, as well as the feasibility of partially replace N fertilizers by liquid or peat-based inoculants containing Ab-V5 cells cultured in Form15.

In fact, *A. brasilense* inoculants containing cells with high amounts of EPS and/or PHB exhibited superior performance in field trials (Fallik and Okon, 1996; Joe et al., 2012). It has been demonstrated that the EPS of *A. brasilense* plays a protective role against extreme temperatures and pH levels and is also related to the anchoring of the bacteria to the root surface (Michiels et al., 1991; Konnova et al., 2001). PHB protects *A. brasilense* against abiotic stresses, delays the decline in its viability and helps the establishment and proliferation of the bacteria in the rhizosphere (Okon and Itzigsohn, 1992; Kadouri et al., 2003). In general, cultures of *A. brasilense* with high amounts of EPS are obtained by growing cells in a culture medium with a high carbon:nitrogen (C:N) ratio, while the culture medium composition and bacterial growth conditions lead to modifications in EPS composition, cell aggregation, flocculation and cyst formation (Fischer et al., 2003). The final composition of Form15 has a low C:N ratio (~12:1) when considering the mean composition of the materials used and its concentration in the culture medium, and this ratio could be even lower (~9:1, similar to the C:N ratio of OAB) by discarding the contribution of sucrose. Nevertheless, such a composition led to the production of bacterial cultures with EPS contents ~3-fold higher than those observed in OAB after 36 h of culture. These values exceeds those observed by using a culture medium developed to favor *A. brasilense* cell aggregation (Burdman et al., 1998), and the specific EPS production was maintained with no decay throughout the experimental period, which was the opposite of other studies that used culture media with high C:N ratios (Bahat-Samet et al., 2004). In addition to the high EPS production and population density, the study of *A. brasilense* Ab-V5 kinetics in Form 15 also indicated a higher growth rate and lower generation time in the log phase (12 h of growth), both in comparison with its growth in the OAB medium used in this study and with the findings of other reports in the literature (Kadouri et al., 2003; Bashan et al., 2011; Trujillo-Roldán et al., 2013).

The variability of the plant response to PGPB inoculation, including bacterial inoculants containing diazotrophic bacteria, is the main argument for the adoption of this technology as an additional practice, instead of its use as an alternative practice aimed to reduce the amount of chemical fertilizers inputs in non-leguminous crops. The results obtained in the greenhouse study demonstrate that the variability and the magnitude of inoculation response are related to the physiological quality of the inoculant bacteria. This can be observed by the growth of maize sown immediately after inoculation of the pots with Ab-V5 grown in different culture media and culturing times, in turn related to different amounts of the EPS and PHB biopolymers. The importance of the physiological quality of the bacterial cells to the reach of inoculant formulations with high efficiency was previously pointed out (Herrmann and Lesueur, 2013; Bashan et al., 2014; Carrasco-Espinosa et al., 2015). In the present study, we demonstrated that the physiology of inoculant cells determines both the plant response to the inoculation and the maintenance of inoculant cells viability in the soil, even in the absence of a host plant. In this sense, the Form 15 culture medium represents an advance for the biomass production of *A. brasilense* Ab-V5 as it provides a rapid and continuous increase in the cell density of the cultures, in addition to promote a high EPS production and PHB accumulation on the cultured cells. These characteristics reflected in a lower influence of the culture time on plant response to inoculation as well as on the viability of inoculant cells in the soil, as can be seen from the correlation coefficients shown in Table 5.

Non-symbiotic diazotrophic bacteria, such as *A. brasilense*, have exhibited direct and indirect evidences of combined nitrogen forms controlling functional activities and the expression of genes, including genes related to nitrogen metabolism (Merrick and Edwards, 1995), PHB synthesis (Sun et al., 2000), and EPS synthesis (Burdman et al., 1998; Hou et al., 2014). In fact, there is evidence that auxin production by *A. brasilense* can be inhibited by combined nitrogen forms (Bar and Okon, 1995; Radwan et al., 2004), which could harm the plant-bacterium interaction and impair or even abolish plant growth-promoting effects (Barbieri and Galli, 1993; Spaepen et al., 2014). Considering this, the use of *Azospirillum* inoculants together with the application of high N fertilizer doses can circumvent many of the benefits provided from the proper use of inoculation technology. This means that great effort should be directed toward clearly defining—at a molecular level—whether the inoculation of non-legume plants with diazotrophic bacteria can be recommended with the use of N fertilization at normal levels or whether lowering the N fertilization inputs can maximize the expression of bacterial growth-promoting traits and the consequent benefits on crop nutrition and productivity.

The efficiency of an inoculant formulated with diazotrophic PGPB depends on chemical, physical and biological characteristics found in the agricultural environment at the time of its application but is always affected by the proper use of a high-quality inoculant formulation (Bashan et al., 2014). We argue that for efficiently inoculation cereal crops with *A. brasilense* without resulting in decreased productivity, reduced N fertilization inputs must be considered. In fact, a meta-analysis of *Azospirillum* inoculation in wheat highlighted that a greater inoculation effect took place with no N fertilization, which corroborates the non-additivity of N fertilization and *Azospirillum* inoculation (Veresoglou and Menexes, 2010). The results presented in this work support the finding that the use of high N fertilization inputs brings no additional benefits to plants inoculated with *A. brasilense* Ab-V5, according to the analysis of two trials in different crop seasons using different maize hybrids cropped each season and regardless of the inoculation treatments tested. This became evident by analyzing the interaction effects to study the simple main effects, as suggested by Schabenberger et al. (2000), which showed that significant effects were masked by the mean interaction value. The separate data analysis aimed to estimate the simple effects of treatments and showed that the inoculation treatments had no effect on most of the studied parameters when N fertilization doses higher than 30 kg N ha^−1^ were used, while the N fertilization effect showed to be significant mostly when applied to uninoculated plants. In addition, in both crop seasons, the inoculation of *A. brasilense* Ab-V5 by treating seeds led to increases in the leaf N content of the plants, suggesting a direct effect of the inoculated bacteria on the plant nutritional status, although we did not address in this study whether this contribution was derived from BNF. Actually, scientific evidence has shown that *Azospirillum* can transfer biologically fixed nitrogen to a host (Bashan and de-Bashan, 2010; de-Bashan et al., 2016) and fully supply the nitrogen demand of *Setaria viridis* when a spontaneous ammonium-excreting mutant was inoculated (Pankievicz et al., 2015).

Moreover, our results demonstrate that obtaining high grain yields is feasible with low N fertilization inputs by using an inoculant formulation based on PGPB cells with a high-quality physiological status that can be delivered in a peat-based or liquid formulation and used as a seed treatment or sprayed as a topdressing. Likewise, Fukami et al. (2016) evaluated alternative inoculation methods for *A. brasilense* and found that the application of inoculants via soil or foliar spray showed the same growth-promoting effect as that observed in plants inoculated by seed treatment. Since commercial maize seeds intrinsically have many chemicals that may interfere with the viability of inoculant bacteria and plant colonization, and as farmers are normally averse to expending the time and effort for extra seed preparations, alternative and efficient methods of PGPB inoculation represent another field of study to strengthen and expand the non-legume inoculation practice. Beyond this, the findings of this study contribute to strengthen the inoculation practice with diazotrophic PGPB in non-leguminous crops, by demonstrating that a proper inoculant formulation favors to the establishment of plant-bacteria association then supporting high yields with reduced use of N fertilizer inputs. This is an important step to move the agricultural practices toward sustainability in order to replace, as far as possible, the chemical inputs produced from non-renewable sources of materials and energy by the beneficial naturally-occurring relationships observed between plants and representatives of soil microbiome, such as the PGPB strains used in commercial inoculants.

## Author contributions

AO: Corresponding author; wrote the manuscript; experimental designs, result analysis. OS, PM, and KM: Experimental design; performed experiments; result analysis; reviewed themanuscript. MZ: Experimental design; Performed experiments; reviewed the manuscript. CZ: Experimental designs; reviewed the manuscript. LG: Result analysis; reviewed the manuscript.

### Conflict of interest statement

The authors declare that the research was conducted in the absence of any commercial or financial relationships that could be construed as a potential conflict of interest.
