# Supplementary material for: Maize Inoculation with Azospirillum brasilense Ab-V5 Cells Enriched with Exopolysaccharides and Polyhydroxybutyrate Results in High Productivity under Low N Fertilizer Input
Source: Front Microbiol. 2017 Sep 26;8:1873. doi: 10.3389/fmicb.2017.01873 (PMC5623045; doi:10.3389/fmicb.2017.01873)
Supplement: Supplementary file 1 [file Table1.DOCX]

Supplementary Material

Maize inoculation with *Azospirillum brasilense* Ab-V5 cells enriched with exopolysaccharides and polyhydroxybutyrate results in high productivity under low N fertilizer input

André Luiz Martinez de Oliveira*^1^, Odair José Andrade Pais dos Santos^2^, Paulo Ricardo Franco Marcelino^3^, Karina Maria Lima Milani^1^, Mónica Yorlady Alzate Zuluaga^1^, Claudemir Zucareli^2^, Leandro Simões Azeredo Gonçalves^2^

*** Corresponding Author:** ALMO, almoliva@uel.br

# Supplementary Table 1. Maize genotypes used in this study and agronomic information of the field trials

| Characteristics | Plant genotype | | |
| --- | --- | --- | --- |
|  | 2B610 | AG 2014 | 2B587Hx |
| Germplasm | Simple hybrid | Double hybrid | Simple hybrid |
| Crop cycle | Early | Early | Early |
| Transgene proteins^a^ | Cry1A.105, Cry2Ab2, Cry1F, EPSPS, AAD-1 | Non-GM maize | Cry1F, PAT |
| Kernel type | Semi-dent | Semi-flint | Semi-dent |
| Kernel color | Yellow-orange | Yellow-orange | Yellow-orange |
| Plant height (m) | 2.3 | 2.5 | 2.05 |
| Ear height (m) | 1.3 | 1.3 | 1.05 |
| Sowing date | 9/01/2017 to 17/01/2017 | 05/11/2010 | 27/10/2012 |
| Spacing (m) | NA^b^ | 0.9 | 0.9 |
| Number of seeds m^-1^ | NA | 6 | 6 |
| Population ha^-1^ | NA | 66,000 | 66,000 |
| Date of harvest | 08/02/2017 to 16/08/2017 | 30/03/2011 | 22/03/2013 |

^a^According to the informations available by the breeder companies. ^b^Not applicable
