# Supplementary figures and images for: Maize Inoculation with Azospirillum brasilense Ab-V5 Cells Enriched with Exopolysaccharides and Polyhydroxybutyrate Results in High Productivity under Low N Fertilizer Input
Source: Front Microbiol. 2017 Sep 26;8:1873. doi: 10.3389/fmicb.2017.01873 (PMC5623045; doi:10.3389/fmicb.2017.01873)

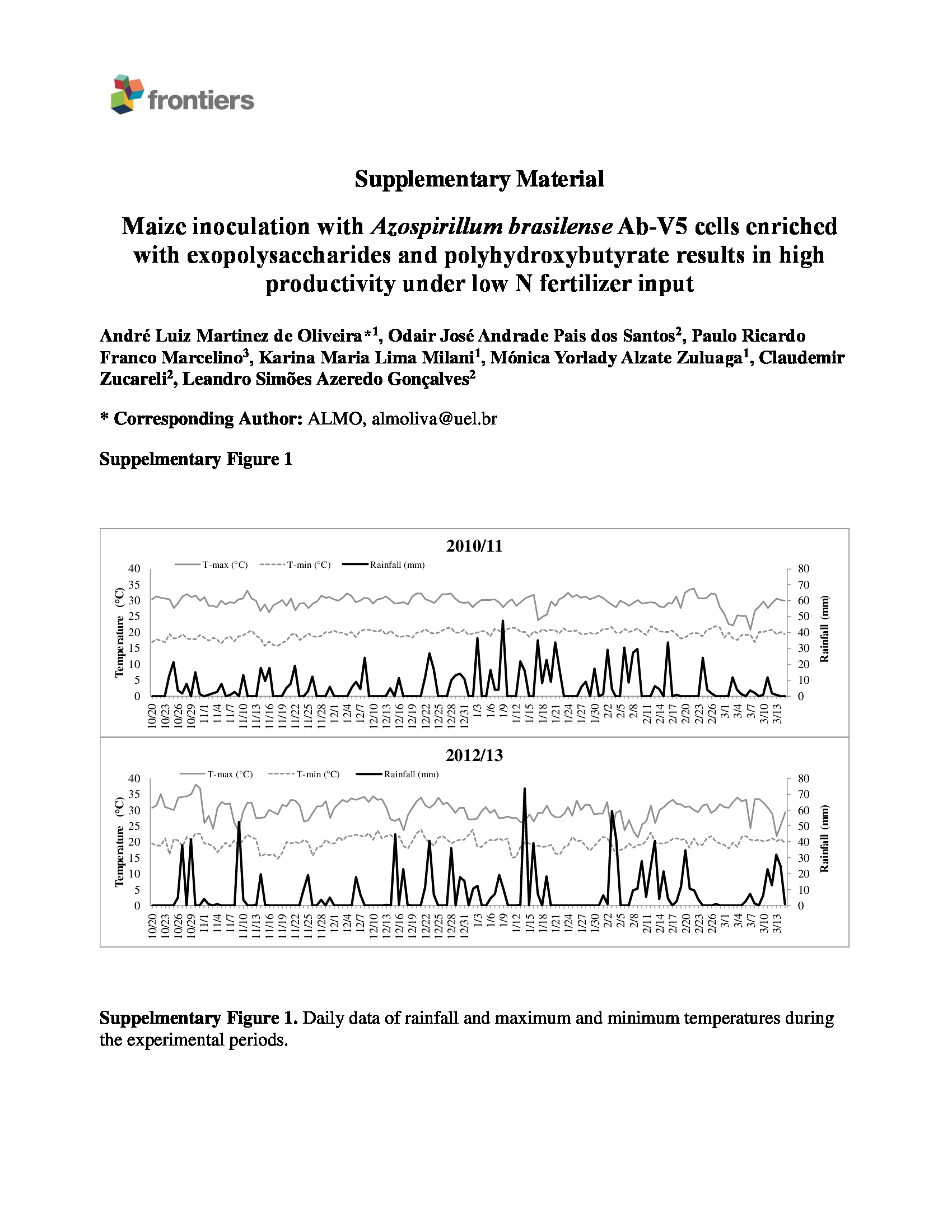

Supplement: Supplementary file 2 [file Image1.JPEG]

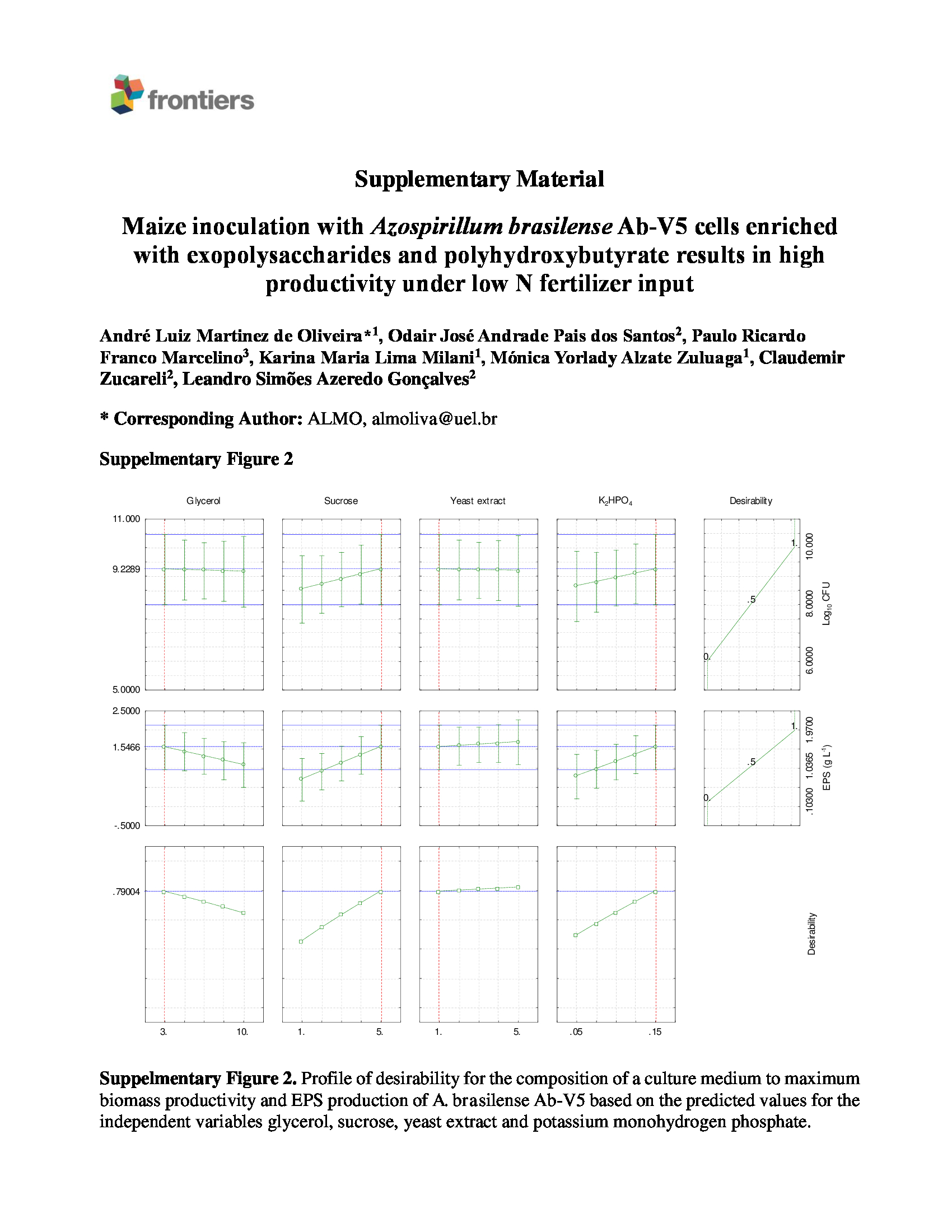

Supplement: Supplementary file 3 [file Image2.JPEG]
